# Differences in infectivity between endosymbiotic *Chlorella variabilis* cultivated outside host *Paramecium bursaria* for 50 years and those immediately isolated from host cells after one year of reendosymbiosis

**DOI:** 10.1242/bio.013946

**Published:** 2015-12-30

**Authors:** Y. Kodama, M. Fujishima

**Affiliations:** 1 Department of Biological Science, Faculty of Life and Environmental Sciences, Shimane University, 1060 Nishikawatsu-cho, Matsue-shi, Shimane 690-8504, Japan; 2 Department of Environmental Science and Engineering, Graduate School of Science and Engineering, Yamaguchi University, Yoshida 1677-1, Yamaguchi 753-8512, Japan

**Keywords:** Auxotrophy, *Chlorella variabilis*, Endosymbiosis, Infectivity, Long-term cultivation, *Paramecium bursaria*

## Abstract

*Chlorella variabilis* strain NC64A is an intracellular photobiont of the ciliate *Paramecium bursaria*. NC64A was isolated from *P. bursaria* nearly 50 years ago and was thereafter cultivated outside the host. This study was undertaken to detect changes in its infectivity to *P. bursaria* and its auxotrophy for growth outside the host induced during long-term cultivation. NC64A can grow in Modified Bold's Basal Medium but not in C medium, whereas another symbiotic *Chlorella variabilis* strain, 1N, that was recently isolated from the host grew in C medium but not in Modified Bold's Basal Medium. With regards infectivity, NC64A in the logarithmic phase of growth showed low infectivity to alga-removed *P. bursaria* cells, whereas those in the early stationary phase showed high infectivity of about 30%. Those in the decay phase of growth showed no infectivity. Results show that NC64A has infectivity, but the infection rate depends on their culture age in the growth curve. Furthermore, NC64A that had been re-infected to *P. bursaria* for more than one year and isolated from the host showed a nearly 100% infection rate, which indicates that NC64A can recover its infectivity by re-infection to *P. bursaria*.

## Abstract (precis)

**Summary:** This study was undertaken to detect changes in infectivity induced during long-term cultivation of *Chlorella variabilis* to alga-free *Paramecium bursaria*, and its auxotrophy for growth outside the host.

## INTRODUCTION

Ciliate *Paramecium bursaria* cells can harbor endosymbiotic *Chlorella* spp. in the cytoplasm, with ∼700 algae per *P. bursaria* (Kodama and Fujishima, 2008). A perialgal vacuole (PV) membrane encloses each alga, which is differentiated from host digestive vacuole (DV) membrane, and the PV membrane protects the alga from the host's lysosomal digestion (Karakashian and Rudzinska, 1981; Gu et al., 2002; Kodama and Fujishima, 2009c). The relationship between *P. bursaria* and the symbiotic alga is a mutual symbiosis with *Paramecium* providing symbiotic algae nitrogen components and CO_2_ (Reisser, 1976, 1980; Albers et al., 1982; Albers and Wiessner, 1985). Conversely, the alga supplies the *Paramecium* photosynthetic products, sugars (Brown and Nielsen, 1974; Reisser, 1986), and oxygen (Reisser, 1980). Irrespective of their mutual relationship, we can maintain the alga-removed aposymbiotic white *P. bursaria* cells and the isolated algae independently. Furthermore, symbiosis between the aposymbiotic *Paramecium* cells and the algae isolated from the alga-bearing symbiotic *Paramecium* cells can be artificially induced by mixing them (Siegel and Karakashian, 1959; Karakashian, 1975). Therefore, the symbiotic relationship between these eukaryotes has been recognized as an excellent experimental system for studying interaction between different cells and the evolution of eukaryotic cells as a result of secondary endosymbiosis. Recently, four important cytological events, which are necessary for establishment of endosymbiosis and timings of each event during the infection process, were clarified in our previous studies (Kodama, 2013; Kodama and Fujishima, 2005, 2007, 2009a,b,c, 2010, 2012a,b; Kodama et al., 2007, 2011). These four cytological events are described below. 1) Within 3 min after mixing, some algae display resistance to the host's lysosomal digestive enzymes in the DV, even when digested algae are also present (Kodama and Fujishima, 2005, 2009c). 2) Within 30 min after mixing, algae in the DV begin budding from the DV membrane into the cytoplasm (Kodama and Fujishima, 2005). 3) Within 15 min after separating from the DV, the DV membrane enclosing a single green *Chlorella* (SGC) differentiates to a PV membrane, protecting the alga from lysosomal fusion (Kodama and Fujishima, 2005, 2009b,c). 4) The alga surrounded by a PV membrane translocates beneath the host cell cortex (Kodama, 2013; Kodama and Fujishima, 2005, 2013; Kodama et al*.*, 2011). Furthermore, the PV appears to localize near the host mitochondria and trichocysts (Fujishima and Kodama, 2012).

Recently, nuclear genome analysis of the symbiotic *Chlorella variabilis* strain NC64A (Blanc et al*.*, 2010) and a transcriptome analysis of *P. bursaria* with and without the symbiotic *C. variabilis* strain 1N have been performed (Kodama et al., 2014). Among the 10,557 transcripts, 6698 expressed differentially between symbiotic and aposymbiotic *P. bursaria* cells to a significant degree (Kodama et al., 2014). However, the nuclear genome of the symbiotic *C. variabilis* NC64A revealed expansions of protein families, which might be related to adaptation to endosymbiosis (Blanc et al., 2010). NC64A was isolated from *P. bursaria* nearly 50 years ago (Karakashian et al., 1968). Therefore, it is expected that the algal infectivity to the alga-removed *P. bursaria* cells or the algal proliferative capacity outside the host cells might be different from the algae that have recently been isolated from the host *P. bursaria*. Thus, we examined the infection process of NC64A to *P. bursaria* and auxotrophy of NC64A outside the host.

## RESULTS

### Effects of incubation medium on NC64A growth

**Fig. 1.**
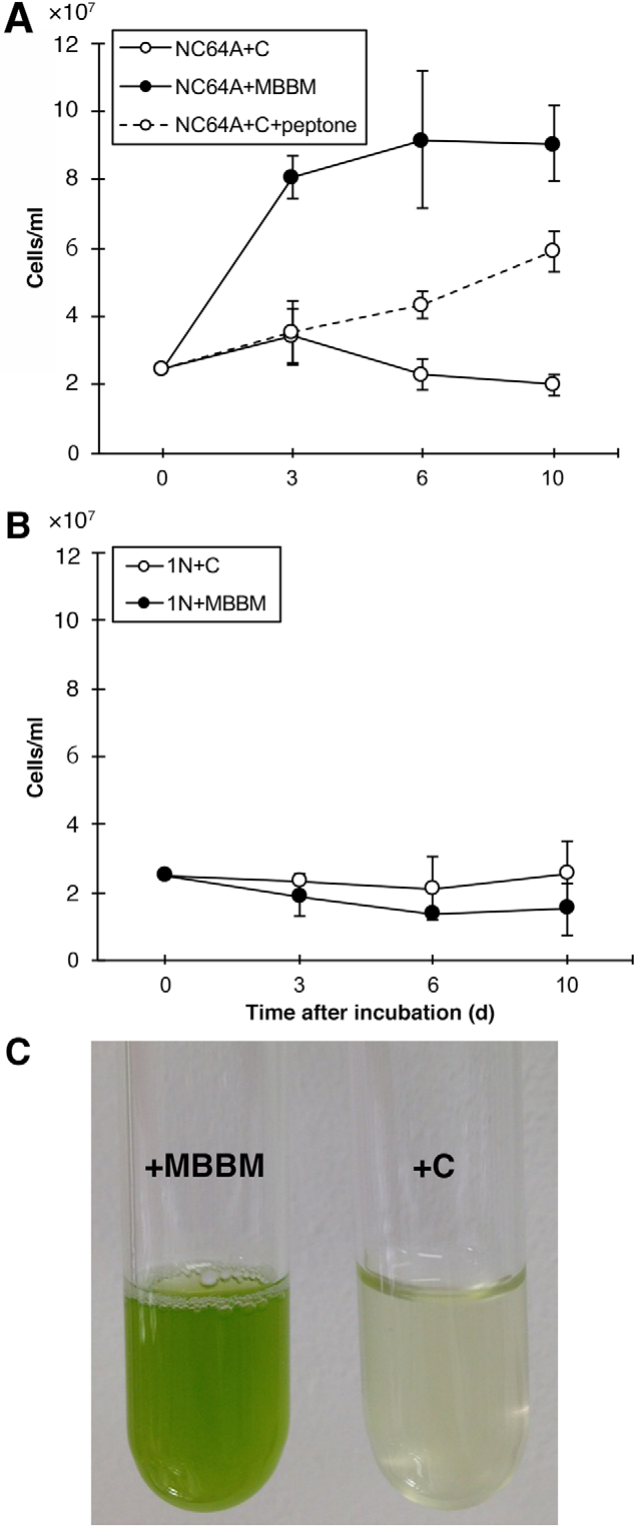
**Effects of the culture medium on strain NC64A growth.** (A) Mean NC64A per 1 ml were counted 3, 6, and 10 days after cultivation in MBBM (closed circle with solid line) and C medium (open circle with solid line) and C medium with peptone (open circle with broken line). (B) Mean 1N per 1 ml were counted 3, 6, and 10 days after cultivation in MBBM (closed circle with solid line) and C medium (open circle with solid line). Vertical bars indicate standard errors of the mean (s.e.m.) for 4–6 samples. (C) Photomicrograph of test tube cultures of NC64A in MBBM (left tube) and in C medium (right tube). These results show that NC64A increased in MBBM but could not increase in C medium. Their growth was rescued by addition of peptone to the C medium. Conversely, 1N increased in C medium as written previously by Kodama et al. (2011) but could not increase in MBBM. The reproducibility of the results was confirmed three times.

*C. variabilis* strain 1N cells isolated from *P. bursaria* strain OS1g1N cells can be cultivated in C medium (Kodama et al., 2011). Blanc et al*.* (2010) cultivated *C. variabilis* strain NC64A cells in Bold's Basal Medium (BBM) modified by the addition of 0.5% sucrose and 0.1% peptone (MBBM) (Agarkova et al., 2008). Therefore, we compared the effects of incubation medium on algal growth. NC64A grew well in MBBM, reaching a stationary phase of growth (approximately 1.0×10^8^ cells/ml) by 6 days after cultivation (Fig. 1A, closed circle with solid line). The cultivated medium showed a bright green color (Fig. 1C, left). By contrast, NC64A was not able to grow in C medium; their number decreased (Fig. 1A, opened circle with solid line and Fig. 1C, right), although their growth in C medium could be rescued by the addition of peptone (Fig. 1A, opened circle with solid line). Conversely, 1N grew faintly in C medium and could not grow in MBBM (Fig. 1B). Table 1 and Table 2 detail the composition of MBBM and C medium, respectively. These results suggest that NC64A requires a large amount of amino acid and sugar for their growth, but 1N does not.

**Table 1.**
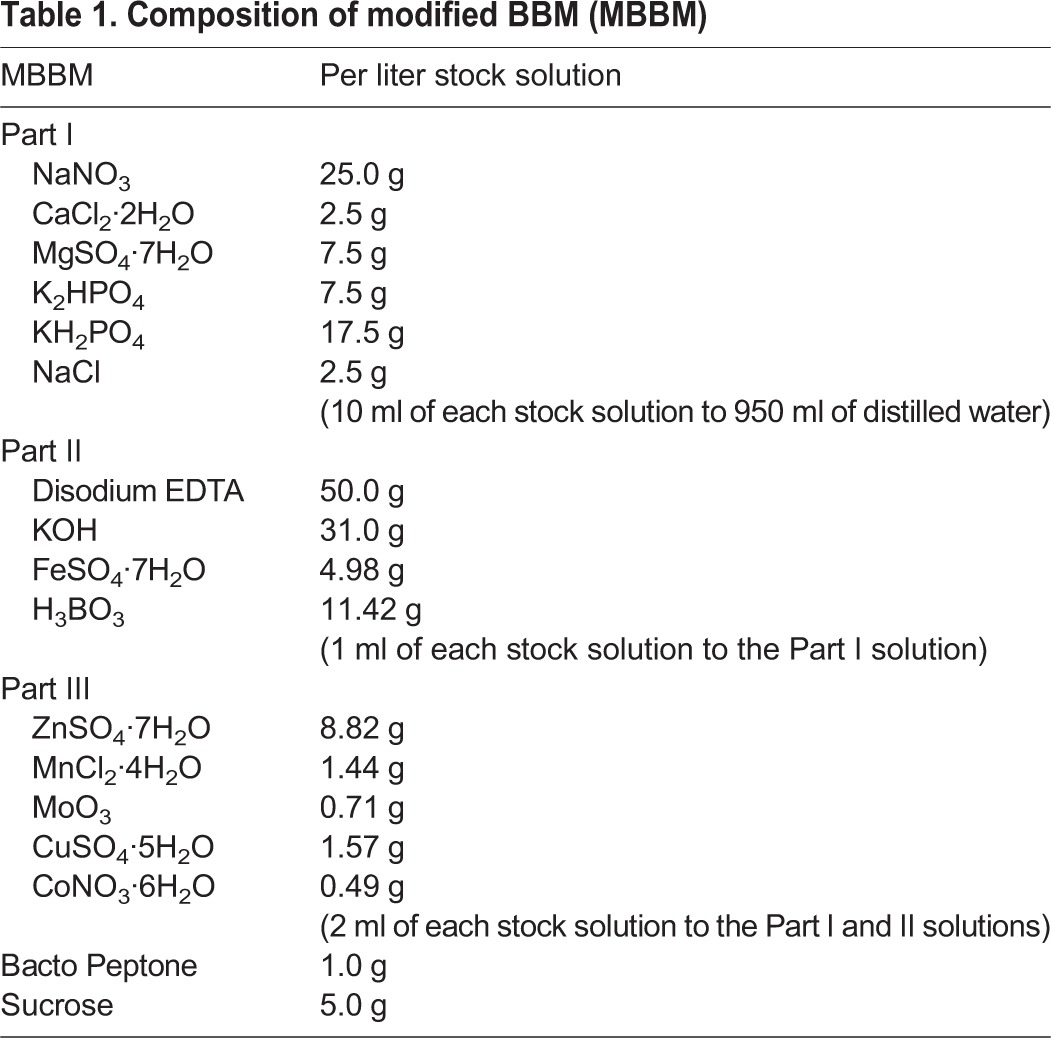
**Composition of modified BBM (MBBM)**

**Table 2.**
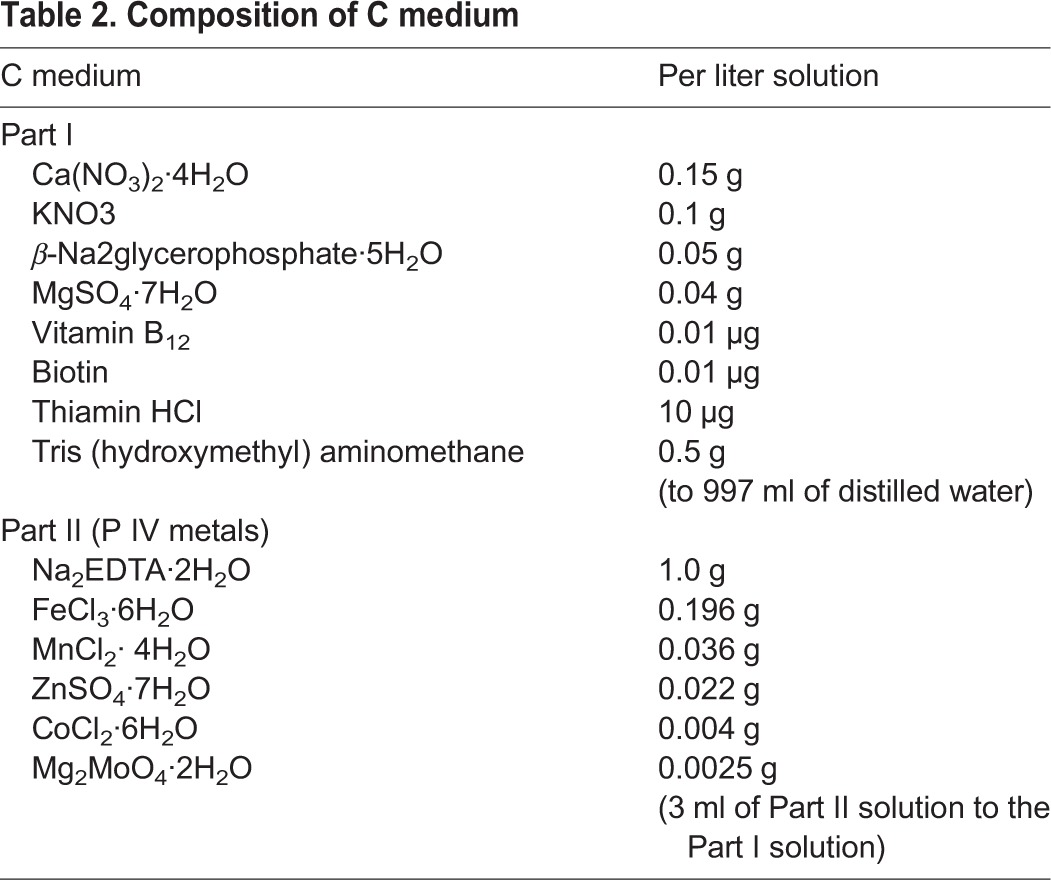
**Composition of C medium**

### Effects of cultivation period on strain NC64A morphology and chlorophyll fluorescence

**Fig. 2.**
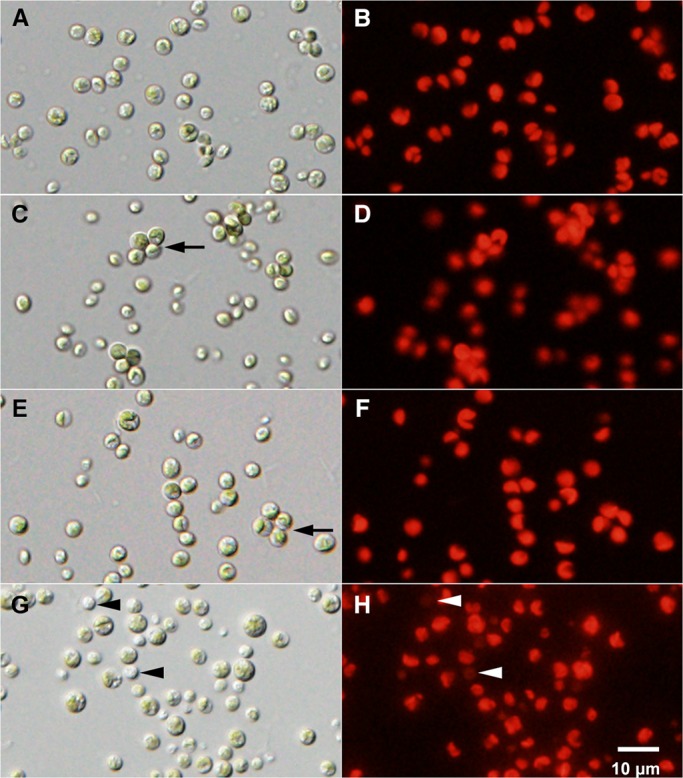
**Photomicrographs of strain NC64A cells cultivated in MBBM.** NC64A cells were observed using DIC (A,C,E,G) and fluorescence microscopy showing autofluorescence of chlorophyll in algal chloroplasts (B,D,F,H) at 1, 4, 8, and 20 days after cultivation. Until 8 days after the cultivation, dividing algae with four autospores were observed (A,C, E), but few were observed at 20 days after the cultivation (G). Similarly, the autofluorescence of chlorophyll, which remained constant until 8 days after the cultivation (B,D,F). The autofluorescence of the colorless algae (G, arrowhead) became weak 20 days after the cultivation (H, arrowhead). Arrows indicate dividing cell with four autospores. The reproducibility of these results was confirmed five times.

Strain NC64A cells were observed by a differential interference contrast (DIC) and fluorescence microscope on days 1, 4, 8, and 20 after cultivation in MBBM. Many mature and immature small cells were observed on day 1 after the cultivation. The algal color was green (Fig. 2A). Many dividing algae with four autospores were observed 4 days after the cultivation, (Fig. 2C, arrow). Small cells were increased soon after the cell division (Fig. 2C). At 8 days after the cultivation, the dividing algae were still visible (Fig. 2E, arrow), and algal color remained green up to 8 days. At 20 days after cultivation, there were few dividing cells, with the majority of the algae as matured large green cells or small colorless cells (Fig. 2G, arrowhead). Fig. 2B,D,F shows that the algal autofluorescence intensity of chlorophyll was not different until 8 days. Colorless cells as shown in Fig. 2G (arrowhead) decreased their autofluorescence 20 days after cultivation (Fig. 2H, arrowhead).

### Infectivity of strain NC64A cells to alga-removed *P. bursaria* cells

**Fig. 3.**
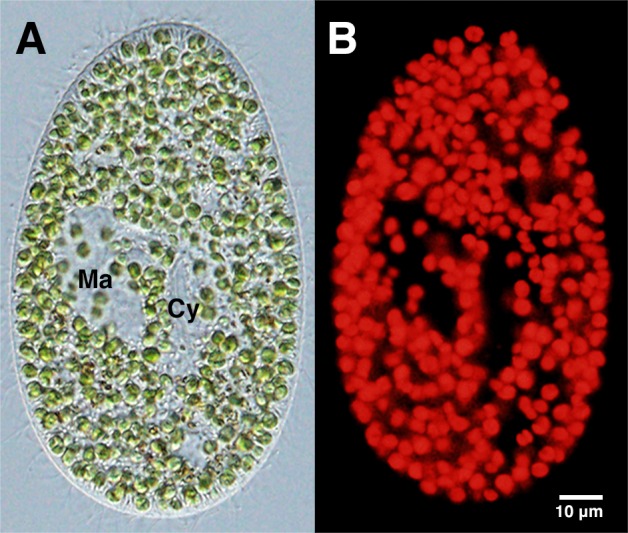
**DIC image and fluorescence microscopy image of strain NC64A-bearing *P. bursaria*.** (A) Many green NC64A are visible in the host cytoplasm in the DIC image, while in (B) autofluorescence of chlorophyll in chloroplast is red. These images show that NC64A is still infective to alga-removed *P. bursaria* cells after 50 years of cultivation outside of the host cells. Ma, Macronucleus; Cy, cytopharynx. Reproducibility of the results was confirmed three times.

One week after the cultivation of NC64A cells in MBBM, their infectivity to *P. bursaria* cells was examined by mixing with aposymbiotic *Paramecium* cells. One week after mixing, the paramecia were observed using DIC and fluorescent microscopy. Soon after mixing with *Paramecium*, many NC64A cells had been taken into many DVs. The timing of DV formation and budding of the DV membrane to release the algae from the DV into the host cytoplasm in this re-infection process were the same as those observed with strain 1N cells (data not shown). Eventually, the NC64A cells localized just beneath the host cell cortex in the same manner as the 1N cells (Kodama and Fujishima, 2005, 2009b, 2011), and established endosymbiosis (Fig. 3A). Autofluorescence of the algal chloroplasts was bright (Fig. 3B). These NC64A cells were transferred stably to daughter cells after subsequent host cell divisions. This stable transfer suggests that timing of cell divisions of both the NC64A cells and the host cell has been well controlled. The NC64A cells were maintained in host cytoplasm more than 1.5 years after mixing with *P. bursaria* cells. The NC64A cells also infected alga-removed *P. bursaria* strain SKK1w cells (data not shown). These results indicate that NC64A cells maintain their infectivity to the aposymbiotic *P. bursaria* cells normally even after 50 years of cultivation outside the host cells.

### Relation between the culture ages of NC64A in MBBM and their infectivity to alga-removed *P. bursaria*

**Fig. 4.**
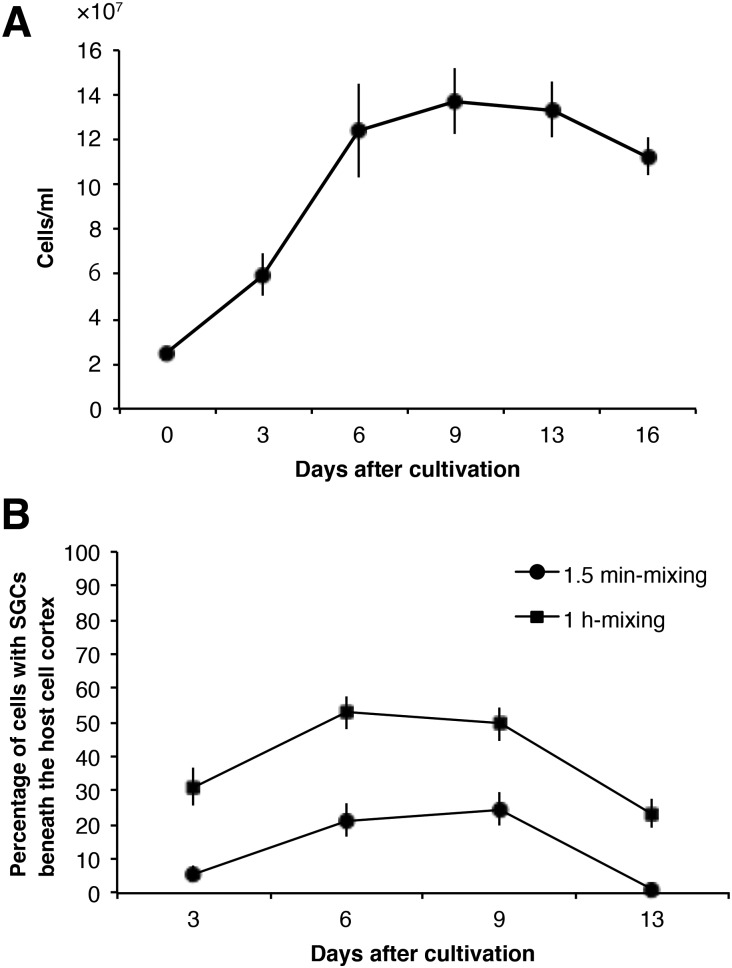
**Growth curve of strain NC64A in MBBM and infectivity of NC64A to alga-removed *P. bursaria* strain Yad1w.** (A) When 2.5×10^7^ cells of strain NC64A were inoculated in 10 ml MBBM, the culture entered early stationary phase and stationary phase at 6 days and 9 days after cultivation, respectively. At 13 days, the culture entered the decay phase and began to decrease the cell density. (B) Infection rates of NC64A to alga-removed *P. bursaria* were examined using two pulse-labeling periods: 1.5 min and 1 h. The infection rates increased gradually in relation to culture ages of the strain NC64A and began to decrease at the decay phase. The same pattern was observed in both pulse-labeling times: 1.5 min and 1 h. Bars in (A) indicate s.e.m. for four samples. Bars in (B) show 90% confidence limits. The reproducibility of the results was confirmed four times.

Fig. 2 shows that algal morphology and intensity of algal autofluorescence changed during cultivation. Therefore, we examined the relation between the culture ages of strain NC64A cells in MBBM (Fig. 4A) and their infectivity to aposymbiotic *P. bursaria* strain Yad1w cells (Fig. 4B)*.* In this experiment, NC64A cells were mixed with the aposymbiotic paramecia for 1.5 min or 1 h because it is known that the algal infection ratio was approximately proportional to the algal density and to the suspension time of paramecia with the algae (Weis and Ayala, 1979). At 3 days, strain NC64A was in the logarithmic growth phase. At this phase, NC64A only slightly infected *P. bursaria* during 1.5 min mixing. With 1 h mixing, about 30% of the paramecia had green algae 24 h after the algal mixing. At six days and nine days after cultivation, strain NC64A cells were in the early stationary phase and stationary phase, respectively. At these phases, the paramecia showed the highest infection ratio (about 20%, 1.5 min mixing; about 50%, 1 h mixing) during the experimental period. At 13 days after the cultivation, the NC64A cells were still in the stationary phase. However, the infection rate was decreased. No NC64A cell remained in paramecia when they were mixed for 1.5 min. By 1 h mixing, although about 25% of paramecia had NC64A cells, the remaining NC64A in the paramecia were few (fewer than 10) and algal color became pale green (data not shown). At 16 days after the cultivation, the NC64A cells were in the death phase. The algal number decreased gradually from that time and the algal color became paler (data not shown). These results show that the infection ratio of NC64A to aposymbiotic *P. bursaria* depends greatly on the stage of their growth curve. The strain NC64A at the death phase had no infectivity.

### Infectivity of strain NC64A cells which had been associated previously with *P. bursaria* cells

**Fig. 5.**
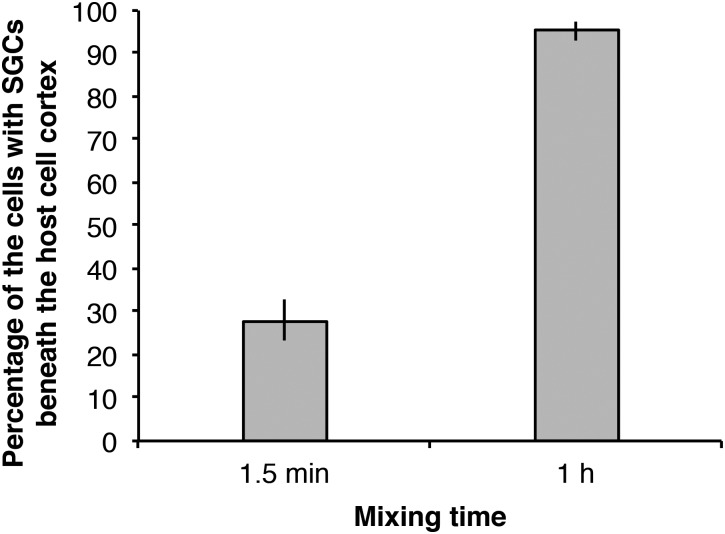
**Infectivity of strain NC64A cells that have been re-infected to *P. bursaria* for more than one year and then isolated from the host cells.** These NC64A cells showed a high infection rate compared to those of NC64A cells cultivated in MBBM without re-infection (Fig. 4B), indicating that the NC64A cells were able to recover their infectivity by re-infection to *P. bursaria*. Bars show 90% confidence limits. The reproducibility of these results was confirmed twice.

**Fig. 6.**
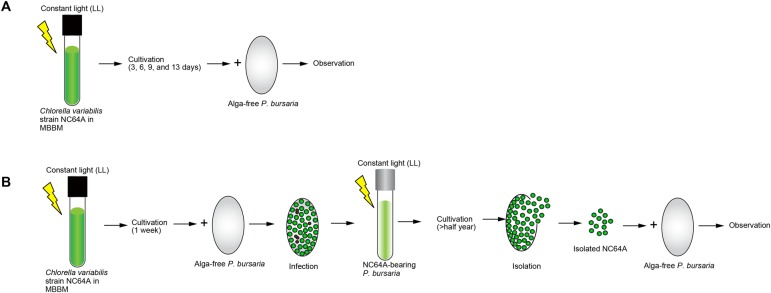
**Schematic representation of the experimental procedures.** (A) Aposymbiotic *P. bursaria,* strain Yad1w cells were mixed with cultivated strain NC64A cells in MBBM for 3, 6, 9, and 13 days at densities of 5×10^3^ paramecia/ml and 5×10^7^ algae/ml for 1.5 min or 1 h at 25±1°C. Strain NC64A-*Paramecium* mixture was washed with MDS, incubated under LL conditions, and observed using DIC microscope with or without fixation. The result obtained using this experiment is presented in Fig. 4. (B) Strain NC64A-bearing *P. bursaria* was established by mixing aposymbiotic *P. bursaria* cells with cultivated NC64A in MBBM; then the strain NC64A-infected *P. bursaria* cells were maintained under LL conditions. Symbiotic strain NC64A cells were isolated from strain NC64A-bearing *P. bursaria*. The algae were mixed with strain Yad1w cells as shown above immediately after the algal isolation. The mixture was washed, incubated under LL conditions, and observed at 24 h after mixing. The result obtained from this experiment is depicted in Fig. 5.

Although the timing of cell divisions of both the symbiotic algae and the host *P. bursaria* is reportedly well coordinated (Kadono et al., 2004; Takahashi et al., 2007), under sufficient feeding conditions the symbiotic algae continue to divide because the symbiotic algal cell division and density are controlled by the nutritional conditions of the host cells (Kodama and Fujishima, 2012a). In this experiment, the infectivity of strain NC64A cells, which had been associated previously with *P. bursaria* cells, was examined. Fig. 6B shows that the NC64A cells were freshly isolated from strain Yad1gNC64A *P. bursaria* cells. This host maintained symbiotic NC64A cells more than one year after infection. As depicted in Fig. 5, by 1.5 min mixing with aposymbiotic *P. bursaria*, about 30% of the paramecia were infected by the NC64A cells at 24 h after mixing with algae. After 1 h mixing, almost all the observed paramecia could establish endosymbiosis with the NC64A cells. Compared with the result of the infection ratio using the NC64A cells cultivated in MBBM for more than 50 years (Fig. 4B), the ratio in Fig. 5 is notably high, which indicates that the decreased infectivity of symbiotic algae NC64A cells grown outside the host cells for more than 50 years can recover if the algae are re-infected to the host *P. bursaria* cells for one year.

## DISCUSSION

Our results showed that symbiotic alga strain NC64A can grow in MBBM but cannot grow in C medium (Fig. 1). Table 1 shows that MBBM contains bacto peptone and sucrose. Kamako et al. (2005) investigated whether bacto peptone and sucrose were necessary for growth of the axenic algal strains that were isolated from symbiotic *P. bursaria* cells. They have maintained these algal strains for more than two years and showed that bacto peptone was necessary, whereas sucrose was not (Kamako et al., 2005). Blanc et al. (2010) discovered an increase in the number of amino acid transporter genes in the NC64A strain genome (35 proteins). Their results indicate that some of these transporters might be expressed in a symbiotic state with host *P. bursaria*. Furthermore, Blanc et al. (2010) discussed that their results are consistent with that of previous study; Kato et al., (2006). Kato et al. (2006) reported that symbiotic *Chlorella*, including NC64A, have an effective system to import amino acids from the host *P. bursaria* and that the algae use the amino acids as a source of nitrogen instead of nitrate. Our results also showed that amino acids are necessary for NC64A growth and that NC64A cannot grow in the absence of an amino acid, such as in C medium.

Davy et al. (2012) reviewed symbiosis between cnidarian/coral and dinoflagellate, and specifically wrote about molecules that are used as signals between the host and symbiont during the initiation of the symbiosis. In their review, they referred to the symbiosis between the freshwater *Hydra* and its symbiotic *Chlorella* because this symbiosis is well studied especially in host–symbiont recognition and phagocytosis processes. Hohman et al. (1982) and McAuley and Smith (1982) showed that successful colonization of the *Chlorella* cells depend on their ability to release the photosynthetic substance, maltose. Therefore, maltose was assumed to be the key molecule as an initiation signal of *Hydra*–*Chlorella* symbiosis. Reportedly, however, there are large amounts of maltose release type and no release type in endosymbiotic *Chlorella* spp. of *P. bursaria.* For example, at low pH, strain 3N813A releases large amounts of maltose. By contrast, it has been reported that strain NC64A does not release maltose in culture (Dorling et al., 1997). Strain NC64A can infect aposymbiotic *P. bursaria*, as shown in this study. Therefore, one can infer that the maltose release ability is not a crucially important feature for symbiotic *Chlorella* spp. of *P. bursaria*.

The symbiotic alga strain NC64A could grow in a culture medium with nitrate as a nitrogen source (Reisser et al., 1988). However, Kamako et al. (2005) showed that the NC64A strain, which they obtained from the ATCC, could not use nitrate. As the reason for these different results, Kamako et al. (2005) suggested that low ability to utilize nitrate was induced by long-term cultivation in a medium without nitrate. With respect to infectivity, NC64A was able to infect to the aposymbiotic *P. bursaria* as shown in this study. These results suggest that some features such as a capacity for nitrate utilization might be lost, but some features such as ability to infect aposymbiotic *P. bursaria* are not lost during long-term cultivation. This study clarified that resistance to host lysosomal digestion of NC64A and their infection ratio depend on their culture ages (Fig. 4). A previous study demonstrated that some proteins of the symbiotic alga, which are synthesized during the algal photosynthesis, are essential to maintain intracellular structures of the alga and the ability of the PV membrane to protect from host lysosomal digestion (Kodama and Fujishima, 2008; Kodama et al., 2011). Our previous study showed that the increased number of acidic vacuoles of the symbiotic *Chlorella* spp. causes preferential digestion by the host lysosomal digestive enzymes during the early infection process (Kodama and Fujishima, 2014). Kuchitsu et al. (1987) showed that the algal vacuoles increase in number at the stationary phase compared with the algae in the log phase. Also, SDS–PAGE showed that the total amount of the algal proteins decreased depending on the cultivation period (Y.K., unpublished data). Transcriptome and proteome analyses of the symbiotic *Chlorella* cells, both those cultivated outside host cells and those freshly isolated from host cells, are necessary in future studies to assess the capabilities for adaptation of the symbiotic NC64A cells to different environments.

## MATERIALS AND METHODS

### Strains and cultures

Experimental procedures are outlined in Fig. 6. An aposymbiotic *P. bursaria* strain Yad1w cell was obtained from the symbiotic strain Yad1g as written in our reports (Kodama and Fujishima, 2009b,c, 2011). A symbiotic strain Yad1gNC64A (syngen 3, mating type I) cell was used to isolate their algae. The Yad1gNC64A cell was produced by mixing of cloned symbiotic *C. variabilis* strain NC64A cells to the aposymbiotic Yad1w cell. *C. variabilis* strain NC64A was obtained from Dr Guillaume Blanc, Aix-Marseille University, France. For culture of *P. bursaria*, red pea (*Pisum sativum*) extract was used (Tsukii et al., 1995) in modified Dryl's solution (MDS) (Dryl, 1959) (KH_2_PO_4_ was used instead of NaH_2_PO_4_·2H_2_O), and a non-pathogenic *Klebsiella pneumoniae* strain 6081 was inoculated one day before use (Fujishima et al., 1990). In ordinary cultures, several hundred paramecia were transferred into a test tube, and 2 ml of fresh culture medium was added on each of the next 12 days. The cultures were in the early stationary phase at one day after the final feeding. All cultures used for present study were in this phase of growth. Symbiotic *P. bursaria* cells were cultivated at 25±1°C under fluorescent lighting (20–30 μmol photons/m^2^/s) using an incandescent lamp. All strains of *P. bursaria* used were provided from Yamaguchi University, with support in part by the National Bio-Resource Project (NBRP) of the Japan Agency for Medical Research and Development (AMED).

NC64A was cultivated with Bold's Basal Medium (BBM) modified by the addition of 0.5% sucrose and 0.1% peptone (MBBM) (Agarkova et al., 2008) or C medium (Ichimura, 1971) at 25±1°C under fluorescent lighting. The compositions of MBBM and C medium are presented in Tables 1 and 2, respectively. In another experiment, 0.1% peptone was added to the C medium.

### Infectivity of strain NC64A to aposymbiotic *P. bursaria* cells

Aposymbiotic *P. bursaria,* strain Yad1w cells were mixed with isolated algae strain NC64A cells or cultivated NC64A cells in MBBM at densities of 5000 paramecia/ml and 5×10^7^ algae/ml for 1.5 min or 1 h at 25±1°C. The cultivated NC64A cells were washed five times with MDS by centrifuging at 4500×***g*** for 1 min at 25°C. The NC64A-*Paramecium* mixture was washed with MDS. The mixture was kept under LL condition. Then, using a differential interference contrast (DIC) and fluorescence microscope (BX51; Olympus Corp.), the *P. bursaria* cells were observed with or without fixation as shown in our previous reports (Kodama and Fujishima, 2005, 2007, 2009a,b,c; Kodama et al., 2007). For fixation, paramecia were mixed with an equal volume of 8% (w/v) paraformaldehyde dissolved in phosphate-buffered saline (PBS; 137 mM NaCl, 2.68 mM KCl, 8.1 mM NaHPO_4_·12H_2_O, 1.47 mM KH_2_PO_4_, pH 7.2). The percentage of paramecia with single green *Chlorella* cells localized under the host cell cortex was determined 24 h after mixing with NC64A cells. This percentage was defined as the infection rate (Kodama and Fujishima, 2005, 2007, 2009b; Kodama et al., 2007). Images were captured digitally using a camera system (Olympus DP73; Olympus Corp.).

### Isolation of alga NC64A cells from symbiotic *P. bursaria* strain Yad1gNC64A cells

Alga NC64A cells were isolated from symbiotic *P. bursaria*, strain Yad1gNC64A cells as previously described (Kodama et al., 2007). The cell density was ascertained using a blood-counting chamber.
